# Differences in Abortion Use by Sexual Orientation in 3 National Cohorts

**DOI:** 10.1001/jamanetworkopen.2025.8644

**Published:** 2025-05-06

**Authors:** Payal Chakraborty, Sarah McKetta, Colleen A. Reynolds, Mikaela H. Smith, Heidi Moseson, Ariel Beccia, Kodiak R. S. Soled, Tabor Hoatson, Aimee K. Huang, A. Heather Eliassen, Sebastien Haneuse, Brittany M. Charlton

**Affiliations:** 1 Department of Population Medicine, Harvard Pilgrim Health Care Institute, Boston, Massachusetts; 2 Department of Epidemiology, Harvard T.H. Chan School of Public Health, Boston, Massachusetts; 3 Division of Epidemiology, College of Public Health, The Ohio State University, Columbus; 4 Ibis Reproductive Health, Oakland, California; 5 Division of Adolescent/Young Adult Medicine, Boston Children’s Hospital, Boston, Massachusetts; 6 Mongan Institute, Massachusetts General Hospital, Boston; 7 Department of Social and Behavioral Sciences, Harvard T. H. Chan School of Public Health, Boston, Massachusetts; 8 Department of Nutrition, Harvard T.H. Chan School of Public Health, Boston, Massachusetts; 9 Channing Division of Network Medicine, Harvard Medical School and Brigham and Women’s Hospital, Boston, Massachusetts; 10 Department of Biostatistics, Harvard T.H. Chan School of Public Health, Boston, Massachusetts

## Abstract

**Importance:**

Sexual minority individuals have less access to high-quality reproductive health care—including contraceptive care—and have higher rates of unintended pregnancies than their heterosexual peers. Little is known about differences in abortion use by sexual orientation.

**Objective:**

To quantify differences in abortion use by sexual orientation.

**Design, Setting, and Participants:**

This study using data from 3 North American cohorts included pregnancies between 1959 and 2024. Nurses’ Health Study II (NHS2) is a cohort of female nurses in the US, Growing Up Today Study (GUTS) is a cohort of NHS2’s offspring, and Nurses’ Health Study 3 (NHS3) is a cohort of nurses and nursing students in the US and Canada.

**Exposure:**

Sexual orientation (completely heterosexual, heterosexual with same-sex experience, mostly heterosexual, bisexual, and lesbian or gay).

**Main Outcome and Measure:**

Participant-reported pregnancy outcome (induced abortion vs any other pregnancy outcome).

**Results:**

Of a total of 235 948 pregnancies (with nonmissing pregnancy outcome data) across 85 640 participants, 211 095 pregnancies (89.5%) were to completely heterosexual participants, and 24 853 (10.5%) were to sexual minority participants. In GUTS and NHS3, there were a higher percentage of pregnancies to sexual minority participants (1546 [17.7%] and 7425 [19.7%], respectively) than in NHS2 (15 882 [8.4%]). In the cohorts combined, 20 243 pregnancies (8.6%) ended with an induced abortion. Compared with pregnancies to completely heterosexual participants, those to sexual minority participants were more likely to end with an induced abortion (risk ratio [RR], 1.93 [95% CI, 1.85-2.02]). Among sexual minority subgroups, heterosexual with same-sex experience (RR, 1.56 [95% CI, 1.47-1.66]), mostly heterosexual (RR, 2.15 [95% CI, 2.03-2.29]), bisexual (RR, 2.84 [95% CI, 2.49-3.23]), and lesbian or gay participants (RR, 2.52 [95% CI, 2.14-2.95]) had higher abortion use.

**Conclusions and Relevance:**

In this study using data from retrospectively reported pregnancies from 3 longitudinal cohorts, all sexual minority groups had increased abortion use compared with completely heterosexual participants, and abortion use was heterogeneous; given the higher use of abortion among sexual minority populations, they are more likely to be disproportionately impacted by the narrowing of abortion access in the US after the Supreme Court *Dobbs* decision. Future research is needed to understand the pathways that contribute to the unique abortion care needs of sexual minority individuals, in order to provide adequate support for abortion seekers.

## Key Points

**Question:**

How does abortion use differ by sexual orientation?

**Findings:**

In this study using data from 3 national cohorts on 235 948 pregnancies among 85 640 participants, all sexual minority groups had increased abortion use compared with completely heterosexual individuals. Abortion use was heterogeneous among sexual minority subgroups, with bisexual and lesbian or gay individuals having the highest abortion use, followed by mostly heterosexual individuals and heterosexual individuals with same sex experience.

**Meaning:**

These findings highlight the need to understand the variability in abortion access among sexual minority individuals, in order to provide adequate support for sexual minority individuals seeking an abortion, especially as abortion access has narrowed since the US Supreme Court *Dobbs* decision.

## Teaser

This cohort study uses data from the Nurses' Health Study 2 and 3 and Growing Up Today Study to assess differences in use of abortion care by sexual orientation.

## Introduction

Emerging evidence indicates that sexual minority individuals (eg, lesbian, gay, bisexual, and queer individuals or those with same-sex or same-gender attractions or partners) are more likely to have unintended pregnancies and induced abortions compared with their heterosexual peers.^1,2^ This trend is similar among other structurally marginalized groups, due to being disproportionately affected by poverty and having less access to high-quality general and reproductive health care—including contraceptive care^3^—and having less access to sex education.^4,5,6^ Furthermore, many sexual minority people live in US states with limited to no abortion access. According to the Williams Institute, 35.9% of lesbian, gay, bisexual, and transgender (LGBT) adults live in the Southern region of the US,^7^ where most states have banned abortion entirely or have severe abortion restrictions (eg, 6-week bans).^8^

Accurate data on sexual orientation differences in abortion use are needed to highlight the reproductive health care needs of sexual minority populations. Abortion surveillance data reported to the Centers for Disease Control and Prevention (CDC) do not include information about sexual orientation, and thus abortion counts by sexual orientation have been unavailable.^9^ A 2023 report by the Guttmacher Institute,^10^ which conducts the only national US survey of abortion seekers with data on sexual orientation, suggested that bisexual, pansexual, and lesbian individuals, or those who have another sexual minority identity, make up over 16% of abortion patients.

However, abortion use among people with other sexual minority identities, such as mostly heterosexual or heterosexual with same-sex or same-gender partners and attractions, remains unknown. Experiences and outcomes across sexual minority subgroups are not monolithic; different sexual minority subgroups have different exposures related to victimization, discrimination, and access to resources, all of which may impact abortion use. For example, bisexual and mostly heterosexual people may experience unique stressors related to biphobia and monosexist discrimination,^11,12^ and heterosexual individuals with same-sex or same-gender partners may experience unique stressors related to dissonance between different sexual orientation dimensions.^13^ These unique stressors may lead to varied abortion care needs. To our knowledge, only 1 study^2^ has examined abortion use across the lifecourse using multidimensional assessments of sexual orientation. This study found that lesbian individuals were less likely to ever have an abortion compared with their completely heterosexual peers, while heterosexual individuals with same-sex partners, mostly heterosexual individuals, and bisexual individuals were more likely to ever have an abortion.^2^ It is unknown if the same trend holds when accounting for multiple abortions per person, precluding our understanding of the true magnitude of inequities.

In this study, we examined differences in abortion use by sexual orientation identity, attractions, and partners using 3 national cohort studies based in North America. To our knowledge, this is the first study to examine differences in induced abortions across diverse sexual orientation groups (ie, completely heterosexual, heterosexual with same-sex experience, mostly heterosexual, bisexual, and lesbian or gay) at the pregnancy level using longitudinal, multidimensional measurements of sexual orientation.

## Methods

### Data Source

We used data from 3 national ongoing cohorts: the Nurses’ Health Study II (NHS2), Growing Up Today Study (GUTS), and Nurses’ Health Study 3 (NHS3). NHS2 is a cohort study of female registered nurses in the US who were enrolled in 1989 from ages 25 to 42 years. Participants completed follow-up questionnaires every 2 years. GUTS recruited the children of NHS2 participants and are followed approximately every 2 years. NHS3 is a cohort of nurses and nursing students living in the US or Canada who were born on or after January 1, 1965. Enrollment in NHS3 started in 2010 and is ongoing. Participants are surveyed every 6 months. In all 3 cohorts, we restricted to participants if they reported having at least 1 pregnancy in their lifetime. Because gender was not measured at enrollment, we use gender-neutral language throughout this manuscript.

We followed the Strengthening the Reporting of Observational Studies in Epidemiology (STROBE) reporting guideline for observational studies. The cohorts were approved by the institutional review boards of Brigham and Women’s Hospital and Harvard T.H. Chan School of Public Health. The present study was a secondary analysis of deidentified data, and was approved by the institutional review board of Harvard Pilgrim Health Care Institute.

### Sexual Orientation

Participants were asked about sexual identity, attractions, and partners multiple times in each cohort (in the 1995, 2009, and 2017 questionnaires in NHS2; nearly every survey in GUTS; and in the fifth, tenth, and thirteenth surveys in NHS3). The sexual identity question was adapted from the Minnesota Adolescent Health Survey, and asked, “Which of the following best describes your feelings?” with response options: completely heterosexual (attracted to persons of the opposite sex), mostly heterosexual, bisexual (equally attracted to men and women), mostly homosexual, completely homosexual (gay or lesbian, attracted to persons of the same sex).^14^ The NHS2 also included a less detailed sexual identity question in earlier questionnaires with only 3 response options: heterosexual, bisexual, and lesbian or gay. For each cohort, we combined the available information to create the following categories: (1) completely heterosexual (reference group), (2) heterosexual with same-sex experience, (3) mostly heterosexual, (4) bisexual, and (5) lesbian or gay (Table 1). Although these sexual orientation groups differ slightly across each cohort depending on the measures that were available, in general, the completely heterosexual group consists of those who identified as completely heterosexual or heterosexual and reported no same-sex or same-gender partners or attractions. The heterosexual with same-sex experience group generally consists of those who identified as completely heterosexual or heterosexual and reported same-sex or same-gender partners or attractions. We analyzed heterosexual with same-sex experience participants as a distinct sexual minority group because they may experience unique minority stressors from discordance between their sexual identity and other dimensions of their sexual orientation, compared with participants who identify with a sexual minority identity and have concordant sexual orientation dimensions (eg, mostly heterosexual or bisexual participants with same-sex partners).^15^ We also examined mostly heterosexual people as a distinct sexual minority group because this group has also been shown to experience unique forms of minority stress; for example, they might experience forms of minority stress similar to bisexual people (eg, identity erasure, less social support).^16^ Additionally, some sources of minority stress may be similar in completely and mostly heterosexual people with same-sex experience; for example, both groups might experience unique internal stressors and discrimination when in a queer-presenting relationship, compared with other sexual minority groups. See eTable 1 and eMethods in Supplement 1 for additional details.

**Table 1.** Summary of Final Sexual Orientation Categories Used for NHS2, GUTS, and NHS3 cohorts

Final categories	NHS2 definition	GUTS definition	NHS3 definition
Completely heterosexual	Identified as completely heterosexual or heterosexual, never reported same-sex attractions or partners, or never identified previously with a sexual minority identity	Identified as completely heterosexual and did not report same-sex partners	Identified as completely heterosexual, never reported a prior sexual minority identity, and never reported having partners who were same-sex, same-gender, or nonbinary nor being attracted to people of the same sex, same gender, or nonbinary gender
Heterosexual with same-sex experience	Identified as completely heterosexual or heterosexual, reported same-sex attractions or partners, or identified previously with a sexual minority identity	Identified as completely heterosexual and reported same-sex partners	Identified as completely heterosexual, reported a prior sexual minority identity, reported having partners who were same-sex, same-gender, or nonbinary or reported being attracted to people of the same sex, same gender or nonbinary gender
Mostly heterosexual	Identified as mostly heterosexual	Identified as mostly heterosexual	Identified as mostly heterosexual
Bisexual	Identified as bisexual	Identified as bisexual	Identified as bisexual
Lesbian or gay	Identified as mostly homosexual, completely homosexual, or lesbian or gay	Identified as mostly homosexual, completely homosexual, or lesbian or gay	Identified as mostly homosexual, completely homosexual, or lesbian or gay

Abbreviations: GUTS, Growing Up Today Study; NHS2, Nurses’ Health Study II; NHS3, Nurses’ Health Study 3.

### Induced Abortion

In NHS2, we used pregnancy data from the 2009 questionnaire, where participants reported all pregnancies across their lifetime. In GUTS, participants reported their lifetime pregnancies in the 2019 questionnaire. In earlier questionnaires, participants were asked prospectively about their recent pregnancies. We used pregnancy data from the 2019 questionnaire when available, and pregnancy data from the prospective collection of pregnancies in prior questionnaires. In NHS3, participants reported their lifetime pregnancy history in the first survey, and all pregnancies since then in the thirteenth survey. In all 3 cohorts, participants were asked detailed questions about each of their past pregnancies, including information about pregnancy outcomes, which also included whether the pregnancy ended with an induced abortion.

### Statistical Analysis

Participants were included in the analysis if they reported at least 1 pregnancy and if they had nonmissing data on sexual orientation identity. Very few participants who met these inclusion criteria were missing data on whether the pregnancy ended in an induced abortion (less than 1.4% for all sexual orientation groups). Given the low proportion of missing data, we performed a complete case analysis.

The unit of all analyses were individual pregnancies. We first computed pregnancy-level descriptive statistics for all variables reporting frequencies and percentages for the outcome, ranges for the years of pregnancy, and means and standard deviations for age at pregnancy. We then fit unadjusted log-linear models to calculate risk ratios (RRs) comparing the proportion of induced abortions (vs all other possible pregnancy outcomes) across sexual orientation groups. Next, we adjusted for year of pregnancy as a categorical variable in increments of 5 years to allow for nonlinearity. We performed minimally adjusted analyses because this study is descriptive, and most related variables (eg, age at pregnancy, health care access) are temporally downstream from sexual orientation, and may (at most) be mediators.^17,18^ Furthermore, adjustment for mediators would block the mechanisms through which heterosexism leads to differences in pregnancy outcomes by sexual orientation and may introduce new collider-stratification bias, which may lead to spurious results.^18,19,20,21^ We only present analyses adjusting for year of pregnancy to account for the changes in sociopolitical climate around abortion and heterosexism from 1959 to 2024; lack of adjustment may underestimate or overestimate abortion use differences depending on the extent to which these policy climates impact abortion use and sexual orientation disclosure (more research is needed to disentangle the effects of specific laws and policies on unmet abortion need and abortion use). To account for multiple pregnancies from a given individual and informative cluster sizes, we used generalized estimating equations (GEE) with robust variance estimation and weights equal to the inverse of the cluster size.^22^ We conducted analyses for each cohort separately and subsequently combined estimates from each cohort by pooling the 3 cohorts. In a sensitivity analysis, we included adjustment for age at pregnancy, as age may impact sexual orientation disclosure and abortion use. However, sexual minority individuals are more likely to have teen pregnancies and pregnancies at later ages,^2,23,24,25^ both of which can make them more likely to get an abortion; thus, some of this association is mediating and adjustment can lead to bias. Finally, we presented the proportion of pregnancies ending in induced abortions by each dimension of sexual orientation (ie, identity, attractions, partners) separately. In supplemental analyses, we presented proportions of pregnancies ending in abortion by each cohort in the period before the US Supreme Court *Roe v Wade* decision legalizing abortion, during the period when abortion was legal at the national level, and after the *Dobbs* decision, which overturned *Roe*. We conducted all analyses using R version 4.2.0 (R Project for Statistical Computing). Significance was determined by 95% CI thresholds and 2-sided *P* < .05.

## Results

Of the 143 580 included participants in NHS2, GUTS, and NHS3, 100 736 participants reported at least 1 pregnancy. After excluding participants with missing data on sexual orientation (of which 14 149 NHS3 participants were excluded because of not yet reaching the fifth survey, where sexual orientation was first asked) and pregnancy outcomes, 85 640 participants were included in the analytic sample (eFigures 1-3 in Supplement 1). These participants had a total of 235 948 pregnancies. Of these pregnancies, 211 095 pregnancies (89.5%) were to completely heterosexual participants, and 24 853 (10.5%) were to sexual minority participants (Table 2). In GUTS and NHS3, there were a higher percentage of pregnancies to sexual minority participants (1546 [17.7%] and 7425 [19.7%], respectively) than in NHS2 (15 882 [8.4%]). The average age at pregnancy across groups and cohorts ranged from 25 to 30 years. Between the 3 cohorts combined, included pregnancies covered 1959 to 2024 (NHS2, 1959-2010; GUTS, 1996-2020; NHS3, 1979-2024).

**Table 2.** Characteristics of Pregnancies in the NHS2, GUTS, and NHS3 Cohorts

Characteristics	Pregnancies, No. (%)					
	Completely heterosexual	Heterosexual with same-sex experience^a^		Mostly heterosexual	Bisexual	Lesbian or gay
**NHS2**						
Total	174 463 (91.6)	10 692 (5.6)	4034 (2.1)		529 (0.3)	685 (0.4)
Age at pregnancy, mean (SD), y	28.9 (5.4)	28.8 (5.8)	29.1 (6.2)		27.4 (6.5)	27.2 (5.5)
Year of pregnancy, range	1959-2010	1964-2010	1965-2008		1963-2007	1965-2007
Induced abortion						
No	159 955 (91.7)	9280 (86.8)	3293 (81.6)		396 (74.9)	563 (82.2)
Yes	13 703 (7.9)	1362 (12.7)	735 (18.2)		131 (24.8)	122 (17.8)
Missing	805 (0.5)	50 (0.5)	6 (0.1)		2 (0.4)	0
**GUTS**						
Total	7221 (82.3)	71 (0.8)	1264 (14.4)		179 (2.0)	39 (0.4)
Age at pregnancy, mean (SD), y	29.1 (3.9)	25.8 (3.7)	29.0 (4.4)		26.2 (4.9)	29.7 (4.8)
Year of pregnancy, range	1996-2020	2000-2018	1997-2019		1999-2019	2001-2019
Induced abortion						
No	6736 (93.3)	51 (71.8)	1089 (86.2)		138 (77.1)	37 (94.9)
Yes	447 (6.2)	19 (26.8)	170 (13.4)		40 (22.3)	2 (5.1)
Missing	38 (0.5)	1 (1.4)	5 (0.4)		1 (0.6)	0
**NHS3**						
Total	30 366 (80.3)	1744 (4.6)	4610 (12.2)		778 (2.1)	330 (0.9)
Age at pregnancy, mean (SD), y	28.8 (5.7)	30.6 (6.1)	28.8 (6.3)		28.7 (6.4)	28.3 (7.1)
Year of pregnancy, range	1980-2024	1982-2024	1979-2024		1985-2024	1982-2022
Induced abortion						
No	27 849 (91.7)	1543 (88.5)	3855 (83.6)		651 (83.7)	269 (81.5)
Yes	2405 (7.9)	194 (11.1)	734 (15.9)		119 (15.3)	60 (18.2)
Missing	112 (0.4)	7 (0.4)	21 (0.5)		8 (1.0)	1 (0.3)

Abbreviations: GUTS, Growing Up Today Study; NHS2, Nurses’ Health Study II; NHS3, Nurses’ Health Study 3.

^a^ In NHS2, the heterosexual with same-sex experience group consists of those who identified as completely heterosexual or heterosexual and also reported past same-sex attractions and/or partners, or identified previously as sexual minority. In GUTS, the heterosexual with same-sex experience group consists of those who identified as completely heterosexual and also reported same-sex partners. In NHS3, the heterosexual with same-sex experience group consists of those who identified as completely heterosexual and also reported same-sex, same-gender, or nonbinary gender partners and/or attractions; had prior same-sex, same-gender, or nonbinary partners and/or attractions; or prior sexual minority identity.

Compared with pregnancies to completely heterosexual participants, those to sexual minority subgroups combined were nearly twice as likely to end with an induced abortion (unadjusted RR, 1.93 [95% CI, 1.85-2.02]) after pooling estimates across the 3 cohorts (Table 3). Compared with completely heterosexual participants, pregnancies to heterosexual participants with same-sex experience were 1.56 times as likely (95% CI, 1.47-1.66) to end in an induced abortion, while those to mostly heterosexual (RR, 2.15 [95% CI, 2.03-2.29]) and lesbian or gay (RR, 2.52 [95% CI, 2.14-2.95]) participants were more than twice as likely to end in an induced abortion. Pregnancies to bisexual participants were almost 3 times as likely to end in an induced abortion (RR, 2.84 [95% CI, 2.49-3.23]). Analyses adjusting for year of pregnancy yielded similar results (Table 3). Sensitivity analyses adjusting for age at pregnancy also yielded similar results (eTable 2 in Supplement 1).

**Table 3.** Estimated Risk Ratios of Induced Abortions by Sexual Orientation in Pregnancies in NHS2, GUTS, and NHS3^a^ Abortions

Characteristics	NHS2, RR (95% CI)		GUTS, RR (95% CI)		NHS3, RR (95% CI)		Combined,^b^ RR (95% CI)	
	Unadjusted	Adjusted^c^	Unadjusted	Adjusted^c^	Unadjusted	Adjusted^c^	Unadjusted	Adjusted^c^
**Overall**								
Completely heterosexual	1 [Reference]	1 [Reference]	1 [Reference]	1 [Reference]	1 [Reference]	1 [Reference]	1 [Reference]	1 [Reference]
Sexual minority^d^	1.90 (1.80-2.00)	1.88 (1.78-1.98)	2.21 (1.87-2.62)	1.93 (1.63-2.27)	1.87 (1.72-2.04)	2.23 (2.05-2.42)	1.93 (1.85-2.02)	2.12 (2.03-2.21)
**Subgroups**								
Completely heterosexual	1 [Reference]	1 [Reference]	1 [Reference]	1 [Reference]	1 [Reference]	1 [Reference]	1 [Reference]	1 [Reference]
Heterosexual with same-sex experience^e^	1.57 (1.46-1.68)	1.55 (1.45-1.66)	4.91 (3.32-7.27)	2.31 (1.55-3.43)	1.38 (1.16-1.65)	2.01 (1.69-2.38)	1.56 (1.47-1.66)	1.56 (1.46-1.66)
Mostly heterosexual	2.38 (2.18-2.60)	2.47 (2.26-2.70)	1.92 (1.59-2.32)	1.83 (1.53-2.20)	1.98 (1.80-2.19)	2.25 (2.04-2.48)	2.15 (2.03-2.29)	2.73 (2.56-2.90)
Bisexual	3.38 (2.76-4.14)	3.01 (2.47-3.67)	3.70 (2.73-5.00)	2.39 (1.74-3.29)	2.17 (1.77-2.66)	2.70 (2.22-3.28)	2.84 (2.49-3.23)	3.37 (2.96-3.84)
Lesbian or gay	2.78 (2.30-3.37)	2.40 (1.98-2.90)	1.00 (0.26-3.77)	0.98 (0.30-3.25)	2.20 (1.63-2.96)	1.92 (1.43-2.58)	2.52 (2.14-2.95)	2.48 (2.12-2.91)

Abbreviations: GUTS, Growing Up Today Study; NHS2, Nurses’ Health Study II; NHS3, Nurses’ Health Study 3.

^a^ Risk ratios were obtained using log-linear models with weighted generalized estimating equations.

^b^ Combined by pooling NHS2, GUTS, and NHS3 data.

^c^ Adjusted for year of pregnancy as a categorical variable in 5-year increments.

^d^ Participants who were heterosexual with same-sex experience; mostly heterosexual; bisexual; or lesbian or gay.

^e^ In NHS2, the heterosexual with same-sex experience group consists of those who identified as completely heterosexual or heterosexual, and also reported past same-sex attractions and/or partners, or identified previously as sexual minority. In GUTS, the heterosexual with same-sex experience group consists of those who identified as completely heterosexual and also reported same-sex partners. In NHS3, the heterosexual with same-sex experience group consists of those who identified as completely heterosexual and also reported same-sex, same-gender, or nonbinary partners and/or attractions, prior same-sex, same-gender, or nonbinary partners and/or attractions, or prior sexual minority identity.

Because bisexual participants had the highest abortion use, we performed tests with pregnancies to bisexual participants as the reference group (eTable 3 in Supplement 1). Bisexual participants were significantly more likely to use abortion than all other sexual orientation groups, except lesbian or gay participants.

In analyses looking at the 3 sexual orientation dimensions (ie, identity, partners, attractions) separately, a higher proportion of pregnancies ended in induced abortions among sexual minority participants across all sexual orientation dimensions in all 3 cohorts, with the exception of pregnancies to lesbian or gay participants in GUTS, likely because this group reported very few pregnancies in GUTS (Table 4). Finally, trends in higher abortion use among sexual minority individuals held even when stratifying pregnancies by time periods that were pre-*Roe* (1959 to 1972), *Roe* (1973 to 2021), and post-*Roe* (2022 to 2024) (eTable 4 in Supplement 1).

**Table 4.** Pregnancies That Ended in Induced Abortions by Identity, Partners, Attractions Closest to Pregnancy in NHS2, GUTS, and NHS3

Characteristics	Pregnancies ending in induced abortion, No. (%)		
	NHS2^a^	GUTS^b^	NHS3
Sexual identity			
Completely heterosexual	13 679 (8.2)	466 (6.4)	2718 (8.2)
Mostly heterosexual	NR	170 (13.5)	734 (16.0)
Bisexual	105 (24.6)	40 (22.5)	119 (15.5)
Lesbian or gay	86 (23.2)	2 (5.1)	60 (18.2)
Same-sex partners			
No	NR	575 (6.9)	2758 (8.3)
Yes	NR	89 (23.4)	713 (17.5)
Same-sex attractions			
No	NR	NR	1512 (7.2)
Yes	NR	NR	956 (12.6)

Abbreviations: GUTS, Growing Up Today Study; NHS2, Nurses’ Health Study II; NHS3, Nurses’ Health Study 3; NR, not reported.

^a^ In NHS2, the measure closest to the pregnancy was used, which relies on the 1995 and 2009 measures with less detailed identity options, heterosexual, bisexual, and lesbian or gay. In 1995 and 2009, sex of partners and attractions were not measured.

^b^ In GUTS, sex of attractions was not measured.

## Discussion

Combining data from 3 North American cohort studies, we found that participants of all sexual minority subgroups were more likely to use abortion compared with their completely heterosexual peers. This finding was consistent across all dimensions of sexual orientation (ie, identity, partners, and attractions) separately.

Research on sexual orientation differences in abortion use is sparse. For example, the Guttmacher Institute published national data showing that 16% of abortion seekers reported a sexual minority identity.^10^ Another study found that, compared with completely heterosexual participants with no same-sex partners, completely heterosexual with same-sex partners, mostly heterosexual, and bisexual participants were more likely have had an abortion in their lifetime, but lesbian participants were not.^2^ Conversely, our study using pregnancy-level data found that pregnancies to all sexual minority subgroups, including lesbian participants, were more likely to end in abortion compared with completely heterosexual participants.

Abortion is necessary reproductive health care, regardless of the reason for seeking it. However, sexual minority people may need abortion care more than heterosexual individuals because of factors rooted in structural-, interpersonal-, and individual-level manifestations of heterosexist stigma and discrimination. Sexual minority people experience structural barriers such as higher rates of poverty, less access to health insurance, and less access to health care, resulting in less access to family planning resources.^26^ Sexual minority people also have less access to sex education, as the existing sex education programs in the US are often not LGBTQ+ inclusive. Additionally, sexual minority people experience barriers to high-quality care across the reproductive health spectrum, including contraception care.^27,28^ They often face stigma and discrimination in reproductive health care settings, and thus may be less likely to seek care and/or to receive care that is sought out.^29,30,31^ Furthermore, providers are not routinely trained to take sexual histories that are LGBTQ+ inclusive and many assume sexual minority patients do not engage in sexual activity that puts them at risk for pregnancy. Therefore, clinicians may not be able to accurately assess their patients’ pregnancy risk.^29^

On average, sexual minority people have an earlier sexual debut compared with their heterosexual peers.^32^ Sexual minority people may have sex that puts them at risk for pregnancy, as a part of their sexual identity development, to avoid stigma, or because of pressure from family or friends to conform to heterosexuality.^33,34,35^ Furthermore, sexual minority people are more likely to experience sexual assault.^36,37,38,39,40^ These factors are connected to higher rates of teen and unintended pregnancies among sexual minority people with capacity for pregnancy.^34,35,41,42,43^

The sexual minority stress model explains how distal (eg, discrimination, violence) and proximal (eg, identity concealment, internalized stigma) stress processes adversely impact the mental and physical health of sexual minority people.^44,45,46,47^ Different sexual minority groups have unique minority stress and discrimination experiences, which may impact their reproductive health experiences and care seeking behaviors. These experiences may lead to varied abortion care needs and outcomes. For example, minority stress contributes to unstable relationship dynamics, making sexual minority people at higher risk of intimate partner violence,^48^ which is associated with higher rates of unintended pregnancy and use of abortion care.^49,50,51,52^ Prior studies have shown that bisexual people in particular experience more victimization and discrimination.^53,54,55,56^ Bisexual and mostly heterosexual people experience unique stressors from monosexist discrimination because they often have partners of a different sex or gender.^11,12^ Among heterosexual individuals with same-sex experience, dissonance between different dimensions of their sexual orientation can lead to negative internalizing processes, which are connected to adverse mental and physical health outcomes.^13,57,58^ Such stressors may be linked to higher rates of unintended pregnancies and thus higher need for abortion care.^1,34,59^ Adverse physical health from stress experienced by minority populations may also lead to higher rates of a range of adverse pregnancy outcomes among sexual minority populations, with specific complications that vary by sexual minority subgroup.^23,58,60,61^ Thus, sexual minority people are more likely to need life-saving abortion care. These varied forms of minority stressors and experiences of discrimination align with our finding that abortion use was heterogenous by sexual minority subgroup.

Conversely, greater use of abortion may indicate that at least some sexual minority individuals have greater access and better ability to navigate the health system to obtain an essential pregnancy-related health care service when needed. Particularly, abortion care is a necessary part of some assisted fertility treatments. Lesbian or gay individuals have a higher use of assisted reproductive technologies compared with other sexual minority subgroups,^62^ and may have more information about and access to abortion care in the event of a nonviable pregnancy. Furthermore, individuals may have various reasons for having an abortion, such as new information about their pregnancies or the pregnant person’s own health, change in relationship status, and change in financial situation. More research is needed on the pathways that contribute to the unique abortion care needs of sexual minority individuals specifically, to provide adequate support for sexual minority abortion seekers.

After the June 2022 ruling in *Dobbs vs Jackson Women’s Health Organization* by the US Supreme Court, substantial parts of the US population have lost access to abortion care.^63^ Being denied a wanted abortion has profound negative financial, social, and health-related consequences for the pregnant person, such as being more likely to experience financial insecurity, being less able to care for existing children, having higher exposure to intimate partner violence, and having poorer mental and physical health.^64,65,66,67,68,69,70^ Additionally, pregnancy options counseling experiences, including abortion and contraception care, may be stigmatizing for sexual minority people in varied ways,^27^ which may shape the type of abortion care sexual minority pregnant people prefer (eg, telemedicine, self-managed, procedural), and delays in seeking abortion care. Given that sexual minority people experience barriers across the reproductive health spectrum, they are more likely to be disproportionately impacted by growing abortion restrictions in a post-*Dobbs* climate, and abortion restrictions are likely to exacerbate, entrench, and compound the reproductive health inequities that sexual minority people already face. However, little research documents their care experiences around pregnancy planning and abortion. Furthermore, although our study was one of the only studies examining abortion use among sexual minority populations, data on abortion use may not capture actual need. Thus, more research is needed on the abortion care needs of sexual minority populations with attention to the heterogenous barriers that sexual minority people may face, including barriers to accessing contraception; experiences in seeking abortion care; access to patient navigation resources around abortion; and preferences around telemedicine, in-facility, and self-managed abortion.

Increased abortion use among sexual minority participants may reflect gaps in access to preventive care, including contraception, and sex education that is sexual minority–inclusive. Addressing causes behind these differences, as well as understanding the unique needs of sexual minority abortion seekers, is more critical now, because the narrowing of abortion access in the US after the *Dobbs* ruling. Professionals must resist heteronormative assumptions about patient pregnancy risks and provide high-quality contraceptive counseling to sexual minority individuals. Health care and public health professionals also must tailor existing resources to meet the unique needs of sexual minority abortion seekers, such as assistance from abortion funds and information about abortion medications by mail.^71,72^ Additionally, in areas where abortion remains legal, clinicians must provide high-quality abortion care to sexual minority patients.

### Strengths and Limitations

Although this study is the largest and one of the few studies looking at sexual orientation differences in abortion use, it has several limitations. First, these cohorts primarily consist of non-Latine^73^ White nurses, nursing students, and their offspring, and therefore may have higher socioeconomic status, have greater access to health care resources, and are more racially homogenous compared with the general population. Thus, this study may underestimate abortion use differences because abortion rates are known to be higher among populations with lower financial resources.^6^ Sexual minority orientations in the source population for this study (ie, NHS2, NHS3, and GUTS) approximate other national datasets,^74,75^ but because the data used for this study are restricted to female participants and are at the pregnancy-level, we were unable to directly compare the representation of sexual minority participants to that of other national estimates. The composition of the eligible sample may overestimate or underestimate rates of abortion use to sexual minority people nationally. Second, abortion use was self-reported in this study. Due to the stigmatization of abortion, abortions are likely underreported. Until sexual orientation is added to existing data sources, such as the CDC abortion surveillance reports, research on abortion care needs of sexual minority populations will have to rely on self-reported data. Furthermore, existing data that do not rely on self-report, such as the Guttmacher report and the CDC surveillance reports only represent people who have had abortions or pregnancies that ended in abortions; our study is one of the only studies that also has data on pregnancies that did not end in abortions, which allowed us to look at the proportion of pregnancies that did end in abortion, and is a major strength of this study.

## Conclusions

In this study using data from retrospectively reported pregnancies from 3 longitudinal cohorts, we found that compared with completely heterosexual participants, sexual minority participants were more likely to use abortion care. Thus, abortion restrictions disproportionately impact sexual minority people. These findings highlight the need to understand the variability in abortion access by sexual orientation and the pathways that contribute to the unique abortion care needs of sexual minority individuals, especially as abortion access has narrowed considerably in the US following the *Dobbs* ruling.

## Notes

Supplement 1. **eTable 1.** Sexual Orientation Questions in NHS2, GUTS, and NHS3 **eTable 2.** Estimated Age-Adjusted Risk Ratios of Induced Abortions by Sexual Orientation in Pregnancies in NHS2, GUTS, and NHS3 **eTable 3.** Estimated Risk Ratios of Induced Abortions by Sexual Orientation in Pregnancies in NHS2, GUTS, and NHS3 With Bisexual Participants (the Group With Highest Abortion Use) as the Reference Group **eTable 4.** Pregnancy Outcomes and Percentage of Pregnancies Ending in an Induced Abortion by Cohort and Time Period **eFigure 1.** Flow Diagram of Inclusion of Participants in the Nurses’ Health Study II **eFigure 2.** Flow Diagram of Inclusion of Participants in the Growing Up Today Study **eFigure 3.** Flow Diagram of Inclusion of Participants in the Nurses’ Health Study 3
eMethods.

eReferences.

Supplement 2.
Data Sharing Statement
