# Supplementary material for: Differences in Abortion Use by Sexual Orientation in 3 National Cohorts
Source: JAMA Netw Open. 2025 May 6;8(5):e258644. doi: 10.1001/jamanetworkopen.2025.8644 (PMC12056570; doi:10.1001/jamanetworkopen.2025.8644)
Supplement: Supplement 1. — eTable 1. Sexual Orientation Questions in NHS2, GUTS, and NHS3 eTable 2. Estimated Age-Adjusted Risk Ratios of Induced Abortions by Sexual Orientation in Pregnancies in NHS2, GUTS, and NHS3 eTable 3. Estimated Risk Ratios of Induced Abortions by Sexual Orientation in Pregnancies in NHS2, GUTS, and NHS3 With Bisexual Participants (the Group With Highest Abortion Use) as the Reference Group eTable 4. Pregnancy Outcomes and Percentage of Pregnancies Ending in an Induced Abortion by Cohort and Time Period eFigure 1. Flow Diagram of Inclusion of Participants in the Nurses’ Health Study II eFigure 2. Flow Diagram of Inclusion of Participants in the Growing Up Today Study eFigure 3. Flow Diagram of Inclusion of Participants in the Nurses’ Health Study 3 eMethods. eReferences. [file jamanetwopen-e258644-s001.pdf]

Supplemental Online Content

Chakraborty P, McKetta S, Reynolds CA, et al. Differences in abortion use by sexual orientation in three national cohorts. *JAMA Netw Open.* 2025;8(5):e258644. doi:10.1001/jamanetworkopen.2025.8644

eTable 1. Sexual Orientation Questions in NHS2, GUTS, and NHS3

eTable 2. Estimated Age-Adjusted Risk Ratios of Induced Abortions by Sexual Orientation in Pregnancies in NHS2, GUTS, and NHS3

eTable 3. Estimated Risk Ratios of Induced Abortions by Sexual Orientation in Pregnancies in NHS2, GUTS, and NHS3 With Bisexual Participants (the Group With Highest Abortion Use) as the Reference Group

eTable 4. Pregnancy Outcomes and Percentage of Pregnancies Ending in an Induced Abortion by Cohort and Time Period

eFigure 1. Flow Diagram of Inclusion of Participants in the Nurses' Health Study II

eFigure 2. Flow Diagram of Inclusion of Participants in the Growing Up Today Study

eFigure 3. Flow Diagram of Inclusion of Participants in the Nurses' Health Study 3

eMethods.

eReferences.

This supplemental material has been provided by the authors to give readers additional information about their work.

© 2025 Chakraborty P et al. *JAMA Network Open*.

eTable 1. Sexual orientation questions in NHS2, GUTS, and NHS3

Cohort	Sexual orientation Dimension	Question	Survey
NHS2	Identity	Whether or not you are currently sexually active, what is your sexual orientation or identity?<br>○ Heterosexual<br>○ Lesbian, gay, or homosexual<br>○ Bisexual<br>○ None of these<br>○ Prefer not to answer	1995, 2009
	Identity	Which one of the following best describes your feelings?<br>○ Completely heterosexual (attracted to persons of the opposite sex)<br>○ Mostly heterosexual<br>○ Bisexual (equally attracted to men and women)<br>○ Mostly homosexual<br>○ Completely homosexual (gay/lesbian, attracted to persons of the same sex)<br>○ Not sure	2017
	Past identity	During your life, have you ever identified yourself as mostly heterosexual, bisexual, or lesbian or gay?<br>○ No<br>○ Yes	2017
	Sex of Partners	During your life, the person(s) with whom you have had sexual contact is (are):<br>○ Male(s)<br>○ Female(s)<br>○ Female(s) and male(s)<br>○ I have not had sexual contact with anyone	2017
	Attractions	During your life, have you ever been sexually attracted to females?<br>○ No<br>○ Yes	2017
GUTS	Identity	Which one of the following best describes your feelings?<br>○ Completely heterosexual (attracted to persons of the opposite sex)<br>○ Mostly heterosexual<br>○ Bisexual (equally attracted to men and women)<br>○ Mostly homosexual<br>○ Completely homosexual (gay/lesbian, attracted to persons of the same sex)<br>○ Not sure	GUTS1: 1999, 2001, 2003, 2005, 2007, 2010<br>GUTS2: 2008, 2011<br>GUTS1 and GUTS2: 2013, 2014, 2015, 2016, 2019
	Sex of Partners	During your life, the person(s) with whom you have had sexual contact is	2001, 2003, 2005,

© 2025 Chakraborty P et al. *JAMA Network Open*.

		(are):<br>○ I have not had sexual contact with anyone<br>○ Female(s)<br>○ Male(s)<br>○ Female(s) and male(s)	2007
	Sex of Partners	During your LIFETIME, have you EVER had sexual contact with a FEMALE?<br>○ No<br>○ Yes	2010
	Sex of Partners	During your life, the person(s) with whom you have had sexual contact (however you define it) is/are:<br>○ No sexual contact<br>○ Female<br>○ Male<br>○ Female and male	2011
	Partners	In the past 12 months, the person(s) with whom you have had sexual contact (however you define it) is (are):<br>○ I have not had sexual contact with anyone<br>○ Female(s)<br>○ Male(s)<br>○ Female(s) and male(s)	2013, 2014, 2015, 2016, 2019
NHS3	Identity	Which one of the following best describes your feelings?<br>○ Completely heterosexual (attracted to persons of the opposite sex)<br>○ Mostly heterosexual<br>○ Bisexual (equally attracted to men and women)<br>○ Mostly homosexual<br>○ Completely homosexual (gay/lesbian, attracted to persons of the same sex)<br>○ Not sure	Module 5, Module 10, Module 13
	Prior identity	During your life, have you EVER identified yourself as mostly heterosexual, bisexual, or lesbian or gay?<br>○ No<br>○ Yes	Module 10
	Prior identity	During your lifetime, have you ever thought of yourself as bisexual, gay, lesbian, or anything other than completely heterosexual/straight?<br>○ No<br>○ Yes	Module 13
	Sex of Partners	During your life, the person(s) with whom you have had sexual contact (however you define it) is (are):<br>○ I have not had sexual contact with anyone<br>○ Female(s)<br>○ Male(s)<br>○ Female(s) and male(s)	Module 5
	Sex of Partners	During your LIFETIME, have you EVER had sexual contact with a FEMALE?	Module 10

© 2025 Chakraborty P et al. *JAMA Network Open*.

		○ No<br>○ Yes<br>During your LIFETIME, have you EVER had sexual contact with a MALE?<br>○ No<br>○ Yes	
	Sex of Attractions	Many NHS3 participants have told us they have felt sexually attracted to other females. During your LIFETIME, have you EVER been sexually attracted to FEMALES?<br>○ No<br>○ Yes	Module 10
	Gender of Partners	During your lifetime, with how many different men have you had sexual contact (however you define it)? Please only count times that were voluntary/consensual; do NOT count times that were not voluntary/consensual such as when sexual contact was forced or against your will.<br>○ 0<br>○ 1<br>○ 2<br>○ 3-5<br>○ 6-10<br>○ 11-14<br>○ 15-24<br>○ 25-34<br>○ 35+<br>During your lifetime, with how many different women have you had sexual contact (however you define it)? Please only count times that were voluntary/consensual; do NOT count times that were not voluntary/consensual such as when sexual contact was forced or against your will.<br>○ 0<br>○ 1<br>○ 2<br>○ 3-5<br>○ 6-10<br>○ 11-14<br>○ 15-24<br>○ 25-34<br>○ 35+<br>During your lifetime, with how many different people with another gender (such as gender fluid, non-binary) have you had sexual contact (however you define it)? Please only count times that were voluntary/consensual; do NOT count times that were not voluntary/consensual such as when sexual	Module 13

© 2025 Chakraborty P et al. *JAMA Network Open*.

		contact was forced or against your will.<br>○ 0<br>○ 1<br>○ 2<br>○ 3-5<br>○ 6-10<br>○ 11-14<br>○ 15-24<br>○ 25-34<br>○ 35+	
	Gender of Attractions	During your lifetime, have you ever been sexually attracted to women?<br>○ No<br>○ Yes<br>During your lifetime, have you ever been sexually attracted to men?<br>○ No<br>○ Yes<br>During your lifetime, have you ever been sexually attracted to people with another gender (such as gender fluid, non-binary)?<br>○ No<br>○ Yes	Module 13

© 2025 Chakraborty P et al. *JAMA Network Open*.

eTable 2. Estimated age-adjusted risk ratios of induced abortions by sexual orientation in pregnancies in NHS2, GUTS, and NHS3[a]

	NHS2		GUTS		NHS3		Combined[b]	
	aRR	(95% CI)	aRR	(95% CI)	aRR	(95% CI)	aRR	(95% CI)
Completely heterosexual	1.00	--	1.00	--	1.00	--	1.00	--
Heterosexual with same-sex experience[c]	1.52	(1.42, 1.62)	2.39	(1.57, 3.63)	1.73	(1.47, 2.02)	1.54	(1.45, 1.63)
Mostly heterosexual	2.32	(2.13, 2.53)	1.78	(1.49, 2.13)	1.79	(1.63, 1.96)	2.07	(1.95, 2.19)
Bisexual	2.58	(2.12, 3.13)	1.97	(1.44, 2.70)	1.85	(1.52, 2.24)	2.28	(2.01, 2.57)
Lesbian/gay	2.26	(1.88, 2.73)	1.11	(0.32, 3.89)	1.68	(1.32, 2.14)	2.04	(1.76, 2.37)

NHS2=Nurses' Health Study II; GUTS=Growing Up Today Study; NHS3=Nurses' Health Study 3; aRR=adjusted risk ratio; CI=confidence interval

[a] Risk ratios were obtained using log-linear models with weighted generalized estimating equations adjusting for age. We note that while we adjusted for age, age may be a mediator because sexual minority individuals are more likely to have teen pregnancies and pregnancies at later ages. There may be mediator-outcome confounding due to exposure and thus, estimates may be affected by collider stratification bias.

[b] Combined by pooling NHS2, GUTS, and NHS3 data

[c] In NHS2, the heterosexual with same-sex experience group consists of those who identified as completely heterosexual or heterosexual, and also reported past same-sex attractions and/or partners, or identified previously as SM. In GUTS, the heterosexual with same-sex experience group consists of those who identified as completely heterosexual and also reported same-sex partners. In NHS3, the heterosexual with same-sex experience group consists of those who identified as completely heterosexual and also reported same-sex/gender or non-binary/another gender partners/attractions, prior same-sex/gender or non-binary/another gender partners/attractions, or prior SM identity.

© 2025 Chakraborty P et al. *JAMA Network Open*.

eTable 3. Estimated risk ratios of induced abortions by sexual orientation in pregnancies in NHS2, GUTS, and NHS3 with bisexual participants (the group with highest abortion use) as the reference group[a]

	NHS2		GUTS		NHS3		Combined[b]	
	RR	(95% CI)	RR	(95% CI)	RR	(95% CI)	RR	(95% CI)
Bisexual	1.00	--	1.00	--	1.00	--	1.00	--
Completely heterosexual	0.30	(0.24, 0.36)	0.27	(0.20, 0.37)	0.46	(0.38, 0.57)	0.35	(0.31, 0.40)
Heterosexual with same-sex experience[c]	0.46	(0.38, 0.57)	1.33	(0.83, 2.13)	0.64	(0.49, 0.83)	0.55	(0.48, 0.63)
Mostly heterosexual	0.70	(0.57, 0.88)	0.52	(0.37, 0.72)	0.91	(0.74, 1.14)	0.74	(0.65, 0.85)
Lesbian/gay	0.82	(0.62, 1.08)	0.27	(0.07, 1.05)	1.01	(0.71, 1.45)	0.89	(0.72, 1.09)

NHS2=Nurses' Health Study II; GUTS=Growing Up Today Study; NHS3=Nurses' Health Study 3; RR=risk ratio; CI=confidence interval

[a] Risk ratios were obtained using log-linear models with weighted generalized estimating equations.

[b] Combined by pooling NHS2, GUTS, and NHS3 data

[c] In NHS2, the heterosexual with same-sex experience group consists of those who identified as completely heterosexual or heterosexual, and also reported past same-sex attractions and/or partners, or identified previously as SM. In GUTS, the heterosexual with same-sex experience group consists of those who identified as completely heterosexual and also reported same-sex partners. In NHS3, the heterosexual with same-sex experience group consists of those who identified as completely heterosexual and also reported same-sex/gender or non-binary/another gender partners/attractions, prior same-sex/gender or non-binary/another gender partners/attractions, or prior SM identity.

© 2025 Chakraborty P et al. *JAMA Network Open*.

eTable 4. Pregnancy outcomes and percentage of pregnancies ending in an induced abortion by cohort and time period

		Pre-*Roe* (1959–1972)			*Roe* (1973–2021)			Post-*Roe* (2022–2024)		
		No abortion (N)	Abortion (N)	% ending in abortion	No abortion (N)	Abortion (N)	% ending in abortion	No abortion (N)	Abortion (N)	% ending in abortion
NHS2	Completely heterosexual	11,470	1,655	13%	147,721	11,664	7%			
	Heterosexual with same-sex experience	775	166	18%	8,453	1,155	12%			
	Mostly heterosexual	211	92	30%	3,066	628	17%			
	Bisexual	47	20	30%	349	110	24%			
	Lesbian/gay	78	23	23%	475	94	17%			
GUTS	Completely heterosexual				6,736	447	6%			
	Heterosexual with same-sex experience				51	19	27%			
	Mostly heterosexual				1,089	170	14%			
	Bisexual				138	40	22%			
	Lesbian/gay				37	2	5%			
NHS3	Completely heterosexual				27,477	2,386	8%	268	3	1%
	Heterosexual with same-sex experience				1,425	185	11%	101	8	7%
	Mostly heterosexual				3,746	719	16%	90	4	4%
	Bisexual				615	118	16%	31	1	3%
	Lesbian/gay				264	60	19%	3	0	0%

N=number; %=percentage

© 2025 Chakraborty P et al. *JAMA Network Open*.

eFigure 1. Flow diagram of inclusion of participants in the Nurses' Health Study II

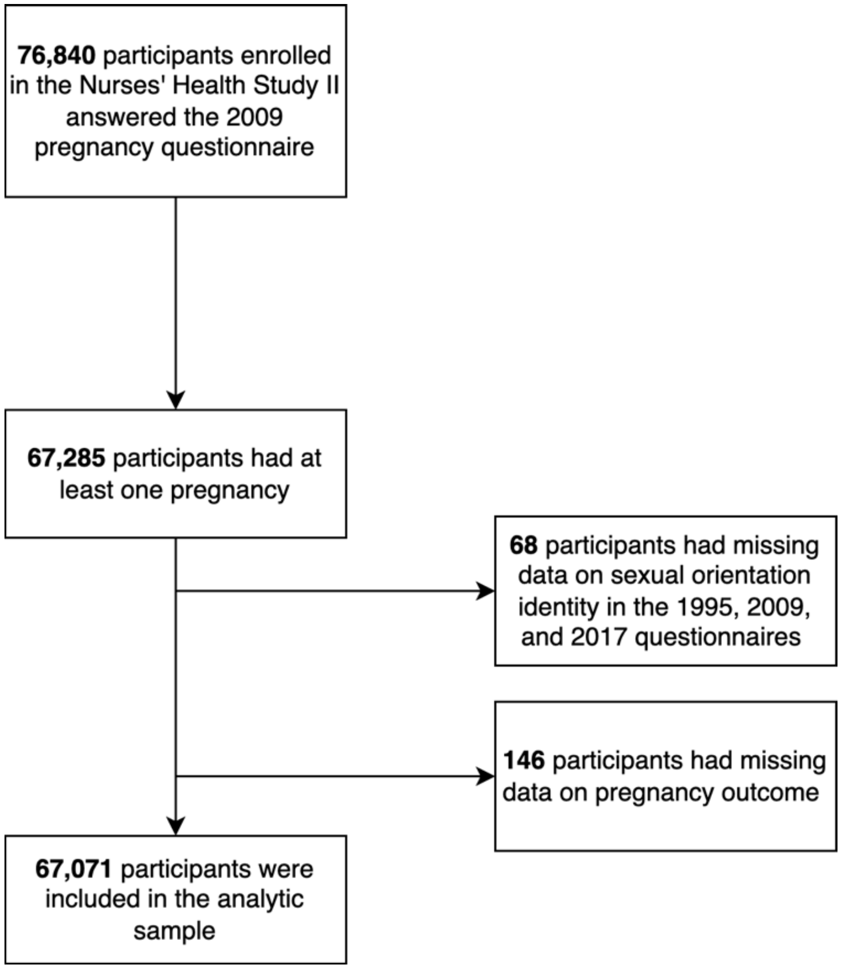

© 2025 Chakraborty P et al. *JAMA Network Open*.

eFigure 2. Flow diagram of inclusion of participants in the Growing Up Today Study

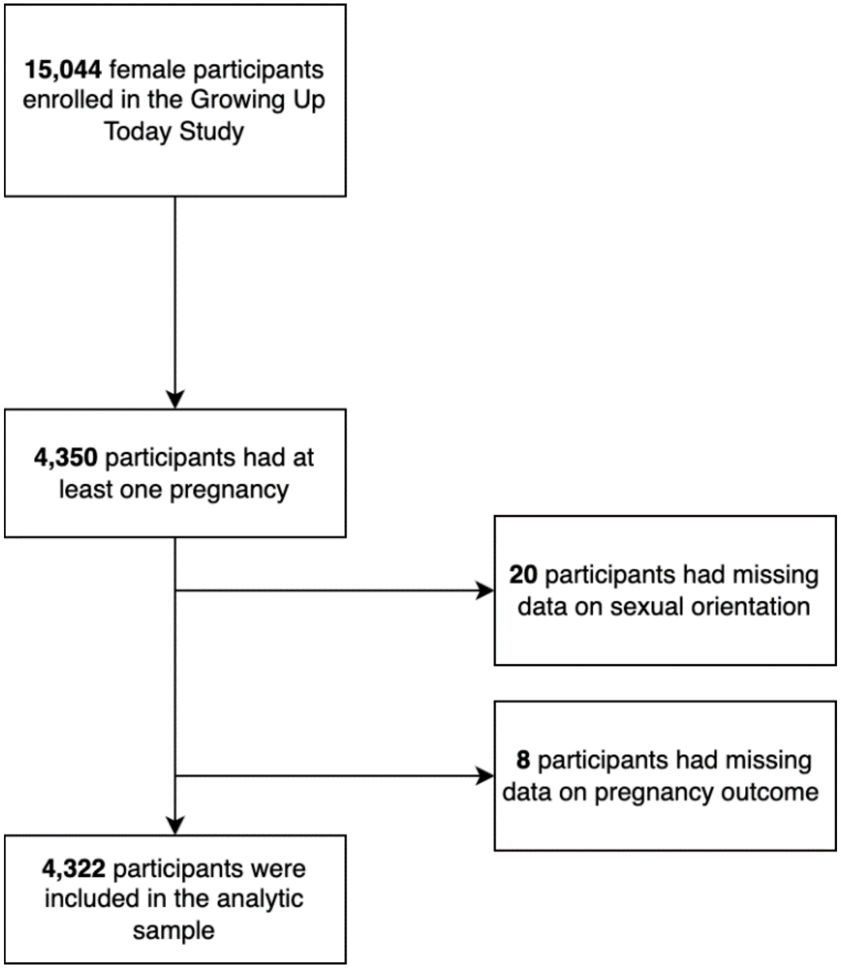

© 2025 Chakraborty P et al. *JAMA Network Open*.

eFigure 3. Flow diagram of inclusion of participants in the Nurses' Health Study 3

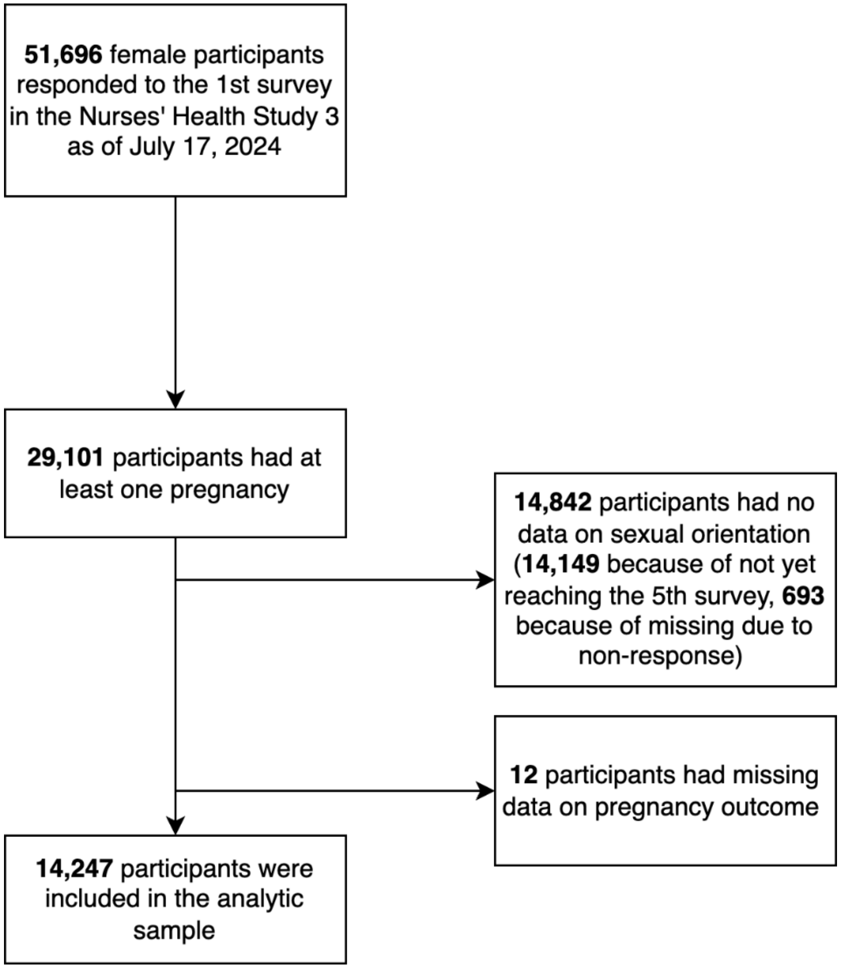

© 2025 Chakraborty P et al. *JAMA Network Open*.

eMethods.

1. Data Sources and Measures

We used data from female participants from three national ongoing cohorts: the Nurses' Health Study II (NHS2), Growing Up Today Study (GUTS), and Nurses' Health Study 3 (NHS3).

1.1. Nurses' Health Study II

The NHS2 is a cohort study of female registered nurses in the U.S., who were enrolled in 1989 when they 25–42 years old. They complete follow-up questionnaires every two years, and the cumulative follow up of the cohort is 88%. NHS2 participants were asked various questions about their health, including reproductive health, chronic health conditions, diet, etc.

In the 2009 survey, participants were asked detailed questions about all of their lifetime pregnancies. For each pregnancy, participants reported the outcome of the pregnancy: single live birth, multiple live birth, stillbirth, miscarriage, tubal/ectopic pregnancy, or induced abortion. We created a dichotomous variable for whether the outcome of the pregnancy was an induced abortion (yes vs. no).

Sexual orientation was measured at three timepoints: 1995, 2009, and 2017. In 1995 and 2009, participants were asked, "Whether or not you are currently sexually active, what is your sexual orientation or identity?" and were given the following identity options: heterosexual; bisexual; or lesbian, gay, or homosexual. In 2017, participants were asked a more detailed identity question adapted from the Minnesota Adolescent Health Survey, which stated, "Which of the following best describes your feelings?" with response options: completely heterosexual (attracted to persons of the opposite sex), mostly heterosexual, bisexual (equally attracted to men and women), mostly homosexual, completely homosexual (gay/lesbian, attracted to persons of the same sex).[1] The 2017 questionnaire also asked additional questions about lifetime sexual orientation identity, sex of sexual partners, and sex of sexual attractions (eTable 1). Because the 2017 variables contain information not just about sexual orientation identity but also attractions and same-sex partners, we primarily used data from this timepoint to construct a lifetime sexual orientation measure. Particularly, if participants were missing identity data in the 2017 questionnaire, we used the most recent identity measure available by (1) combining heterosexual from the 1995/2009 measures with completely heterosexual from the 2017 measure, (2) combining bisexual from the 1995/2009 measures with bisexual from the 2017 measure, and (3) combining lesbian, gay, or homosexual from the 1995/2009 measures with mostly or completely homosexual from the 2017 measure. We then combined this identity measure with data on lifetime identity, attractions, and partners. In summary, we created the following categories for sexual orientation:

- Completely heterosexual or heterosexual, never reported same-sex attractions/partners or never identified previously with a sexual minority identity (shortened name: completely heterosexual)
- Completely heterosexual or heterosexual, reported same-sex attractions/partners or identified previously with a sexual minority identity (shortened name: heterosexual with same-sex experience)
- Mostly heterosexual
- Bisexual
- Mostly or completely homosexual (shortened name: lesbian/gay)

We note that if participants reported a completely heterosexual or heterosexual identity, and were missing data on whether they had past same-sex attractions or partners, or past sexual minority identity, they were included in the reference group.

Because we used a lifetime measure of sexual orientation in the NHS2 cohort, we created a separate measure that was closest to the pregnancy. Because the less detailed 1995 and 2009 sexual orientation measures occurred closer to the time of the pregnancies, these were the measures that were used resulting in the following categories:

- Heterosexual
- Bisexual
- Lesbian/gay

1.2. Growing Up Today Study

GUTS is a cohort of the children of NHS2 participants. The first phase of enrollment (GUTS1) occurred in 1996, enrolling 16,882 children aged 9-14 years. An additional 10,923 children aged 10-17 years were recruited in a

© 2025 Chakraborty P et al. *JAMA Network Open*.

second phase of enrollment (GUTS2) in 2004. Beginning in 2013, both cohorts were combined and followed up together, and have been surveyed every 1-2 years.

Participants were asked about all their pregnancies throughout their lifetime in the 2019 questionnaire. In earlier questionnaires, participants were asked prospectively about their recent pregnancies. We used pregnancy data from the 2019 questionnaire when available, and pregnancy data from the prospective collection of pregnancies in prior questionnaires. Similar to NHS2, GUTS participants reported detailed information about the outcome of each of their pregnancies, including whether the pregnancies ended as a single live birth, multiple live birth, stillbirth, miscarriage, tubal/ectopic pregnancy, or induced abortion. We created a dichotomous variable for whether the outcome of the pregnancy was an induced abortion (yes vs. no).

In GUTS, sexual orientation identity and sex of partners were measured in almost every questionnaire: in 1999 (identity only), 2001, 2003, 2005, 2007, and 2010 for GUTS1 participants; 2008 and 2011 for GUTS2 participants; and 2013, 2014, 2015, 2016, and 2019 for all participants. Because of the availability of multiple measurements, we used the measurement of sexual orientation closest and prior to the pregnancy starting with the 2001 measure (since the 1999 measure only included sexual orientation identity). The identity item in GUTS was the same item adapted from the Minnesota Adolescent Health questionnaire that was used in the 2017 NHS2 questionnaire. We combined this identity measure with information about sex of participants' partners to obtain the following categories:

- Completely heterosexual with no-same sex partners (shortened name: completely heterosexual)
- Completely heterosexual with same-sex partners (shortened name: heterosexual with same-sex experience)
- Mostly heterosexual
- Bisexual
- Mostly or completely homosexual (shortened name: lesbian/gay)

1.3. Nurses' Health Study 3

NHS3 is a cohort of nurses and nursing students living in the U.S. or Canada who were born on or after January 1, 1965. Enrollment in NHS3 started in 2010. Unlike NHS2 and GUTS, enrollment was completed at the same time and participants are given the same survey at the same time, NHS3 is a cohort with open enrollment and participants can be enrolled anytime. The current analysis reflects data available as of July 17th, 2024. Once a participant enrolls, they are asked to complete a sequence of surveys every 6 months. Thus, not all participants complete surveys at the same time. Similar to NHS3 and GUTS participants, NHS3 participants are asked various questions about their health on each survey.

At the time of enrollment on the 1st survey, participants reported all of their lifetime pregnancies. Then at the 13th survey, participants reported all of their lifetime pregnancies since the start of the study when they received the 1st survey. Like NHS2 and GUTS, NHS3 participants were asked about the outcomes of each pregnancy, including whether they ended in a single live birth, multiple live birth, stillbirth, miscarriage, tubal/ectopic pregnancy, or induced abortion. We again created a dichotomous variable for whether the outcome of the pregnancy was an induced abortion (yes vs. no).

In NHS3, sexual orientation was measured in the 5th, 10th, and 13th surveys. In the 5th survey, participants were asked about sexual orientation identity and partners. In the 10th and 13th surveys, participants were asked about sexual orientation identity, attractions, and partners. Again, the identity measure was the same as the measure used in GUTS and the 2017 NHS2 survey. The 13th survey additionally included information about the gender of participants' attractions and partners. Because of the availability of three measurements of sexual orientation, we used the measure closest to the pregnancy. We created the following sexual orientation categories:

- Completely heterosexual, never reported a prior sexual minority identity, and never reported having partners who were same-sex/gender or non-binary nor being attracted to people of the same-sex/gender or non-binary gender (shortened name: completely heterosexual)
- Completely heterosexual, reported a prior sexual minority identity, reported having partners who were same-sex/gender or non-binary, or reported being attracted to people of the same-sex/gender or non-binary gender (shortened name: heterosexual with same-sex experience)
- Mostly heterosexual
- Bisexual
- Mostly or completely homosexual (shortened name: lesbian/gay)

© 2025 Chakraborty P et al. *JAMA Network Open*.

1.4. Summary of Measures in NHS2, NHS3, and GUTS

In each cohort, we created a dichotomous measure for whether participants' pregnancies did or did not end in an induced abortion. Our final sexual orientation categories for each cohort were: completely heterosexual, heterosexual with same-sex experience, mostly heterosexual, bisexual, and lesbian/gay. We used these measures to examine differences in pregnancies ending in induced abortions by sexual orientation.

2. Missing Data

Missingness was low in all three cohorts. Missing data for sexual orientation was low. Only 0.01% of pregnancies were excluded due to missing data on sexual orientation in NHS2, and 0.9% were excluded due to missing data on sexual orientation in GUTS. In NHS3, since enrollment is ongoing, not everyone who reported lifetime pregnancies in the 1st survey have yet progressed to the 5th survey, when sexual orientation was first asked. Therefore, among pregnancies of NHS3 participants who progressed to at least the 5th survey, only 4.5% were excluded due to missing data on sexual orientation. Thus, very few participants' pregnancies in all three cohorts were excluded because of missing data on sexual orientation. Furthermore, very few pregnancies were missing data on the study outcome: whether the pregnancy ended in an induced abortion (ranging from 0% to 1.4% for all sexual orientation groups). Given the low proportion of missing data, we performed a complete case analysis.

© 2025 Chakraborty P et al. *JAMA Network Open*.

eReferences

1. Remafedi G, Resnick M, Blum R, Harris L. Demography of sexual orientation in adolescents. *Pediatrics*. 1992;89(4 Pt 2):714-721.

© 2025 Chakraborty P et al. *JAMA Network Open*.
