# Supplementary material for: Differences in Abortion Use by Sexual Orientation in 3 National Cohorts
Source: JAMA Netw Open. 2025 May 6;8(5):e258644. doi: 10.1001/jamanetworkopen.2025.8644 (PMC12056570; doi:10.1001/jamanetworkopen.2025.8644)
Supplement: Supplement 2. — Data Sharing Statement [file jamanetwopen-e258644-s002.pdf]

Data Sharing Statement

Data
Data available: No

Additional Information
Explanation for why data not available: The data that support the findings of this study are available on request from the Channing Division of Network Medicine at Brigham and Women's Hospital and Harvard Medical School. The data are not publicly available due to privacy or ethical restrictions.
